# Predicting Student Engagement: The Role of Academic Belonging, Social Integration, and Resilience During COVID-19 Emergency Remote Teaching

**DOI:** 10.3389/fpubh.2022.849594

**Published:** 2022-03-18

**Authors:** Melissa Versteeg, Rutger F. Kappe, Carlijn Knuiman

**Affiliations:** Research Group Study Success, Department Education and Innovation, Inholland University of Applied Sciences, Haarlem, Netherlands

**Keywords:** resilience, engagement, academic belonging, social integration, higher education, students, emergency remote teaching

## Abstract

**Background:**

The COVID-19 pandemic has forced higher education (HE) to shift to emergency remote teaching (ERT), subsequently influencing academic belonging and social integration, as well as challenging students' engagement with their studies.

**Aims:**

This study investigated influences on student engagement during ERT, based on student resilience. Serial mediation analyses were used to test the predictive effects between resilience, academic belonging, social integration, and engagement.

**Methods:**

The Student Well-being Monitor (SWM 2021) was completed by 1332 HE students studying at Inholland University of Applied Sciences in the Netherlands. Predictive models were compared among students with low, normal, or high resilience using SPSS extension Macro PROCESS.

**Results:**

A significant serial mediation model was found among all HE students, including positive mediating effects of academic belonging and social integration. More so, independent partial predictive effects of academic belonging and social integration on engagement were also present. Assessment of student resilience profiles revealed substantial differences between predictive models. For low resilience students, serial mediation was present and included the largest partial predictive effect from social integration compared to other groups. For highly resilient students, mediation via academic belonging was found, including the strongest partial and indirect effects compared to other groups.

**Conclusions:**

Overall, academic belonging and social integration positively mediate the effect of resilience on engagement in addition to demonstrating independent positive predictive effects. Inspection of resilience profiles reveals substantial model fit differences, suggesting use of different engagement strategies between student groups. Findings contribute to understanding of HE student engagement during ERT in the Netherlands and provide novel insight on the mechanisms between resilience and engagement. While ERT continues to be required, engagement may be enhanced by stimulating academic belonging for all students generally, but low resilience students could be best served by additionally targeting social integration and resilience.

## Introduction

The rapid transition toward emergency remote teaching (ERT) during the COVID-19 pandemic has had major impacts on student life (1–6). Implemented among higher education institutes (HEIs) as a strategy to curb the spread of the corona virus (7), ERT has been found to coincide with decreased quality of education, negative reactions to online learning, psychological distress, and feelings of uncertainty (8–15). Lack of interaction with teachers and students, prolonged negative mood, and fear of academic delay are impacting higher education (HE) students' well-being and learning (9, 11, 16–18). To date, no detailed study has examined wellbeing and student success during ERT among HE students in the Netherlands.

Engagement is an important precursor of student success (19–21), with studies linking engagement to academic achievement and adaptive coping styles (19, 20). Moreover, significant relationships exist between engagement and wellbeing aspects such as burn-out, depression, and anxiety (6, 9, 22–26). Engagement of HE students can be conceptualized as an enduring and widespread affective-cognitive state (27) including subcomponents of vigor, absorption, and dedication (23, 28–33). Vigor entails a willingness to invest in academic studies, dedication regards a sense of enthusiasm toward studying, and absorption involves becoming engrossed or absorbed by study materials when studying (23).

Engagement has also been found to depend on students' learning environments and interactions with fellow students and teachers (19, 34). The academic environment influences students' sense of belonging, including feeling accepted and valued, and whether they ‘fit’ with their environment (35–37). Moreover, sense of belonging is described as an essential psychological human need, which can be extrapolated to the need to belong within educational settings (38). The level by which individuals experience belongingness within educational settings affects engagement, the quality of social interactions with peer students, and academic performance (39, 40).

Social integration plays a significant direct and indirect role in engagement too (40, 41), as studies positively link students' sense of belonging to social interactions with fellow students and engagement (42–44). Students who feel like they do not belong will extend such beliefs into their social behaviors, risking isolation from peer students and risking reduced academic success (38, 39). A study even reported the strongest predictive effect between students' social interactions and engagement levels (41). Although ERT research has investigated changes in engagement related to study activity dynamics, student mindsets, and technology use among students (3, 12, 45), it has yet to examine belongingness and social integration during ERT. With students reporting increased isolation (46) and decreased quality of student social interactions (47) during the pandemic, investigation into these variables is called for.

Resilience is also pivotal to engagement, academic achievement, and wellbeing (24, 38, 43–55), and is defined as an individual's ability to bounce back following stress exposure (56). Resilience is furthermore deemed critical to maintained mental wellbeing throughout stressful experiences linked to the COVID-19 pandemic (13, 57). The increased experience of stress among students during this time (58, 59), may thus be navigated more successfully by sufficiently resilient students, and positive relationships between student resilience and engagement have been reported under non-ERT conditions (60, 61). More so, a recent study on teacher resilience during ERT did show significant associations between maintenance of teaching quality and resilience during ERT (62), though no studies on resilience and engagement among HE students during ERT exist currently.

ERT differs from online distance learning as ERT is developed to provide educational access during an emergency or crisis and is quick to set up, focused on short-term solutions, and pays little attention to design (63–65). As a consequence, engagement during ERT involves specific challenges, with engagement linked to adaptivity to online teaching, attendance, emotional states, and teaching strategies (6, 10, 16, 18). Studies have yet to analyse student resilience profiles related to engagement during ERT. As a result, the current study elected to examine the relationships between these constructs among students studying via ERT at HEIs.

With research on face-to-face learning indicating positive directional associations between resilience, engagement, belongingness and collaborations with fellow students (24, 35, 37–40, 49–51, 60), we also expect positive predictive effects among these constructs in HE students during ERT. Regarding group differences, expectations draw from findings indicating that resilient students utilize their academic environment effectively (50), and successfully use social relationships with peers to promote engagement (41, 43). Furthermore, given study outcomes indicating that highly resilient individuals can ‘thrive’ during stress exposure (56, 66), we expect the predictive model effects to be lowest among HE students with low resilience, and highest among highly resilient HE students.

**Figure 1:**
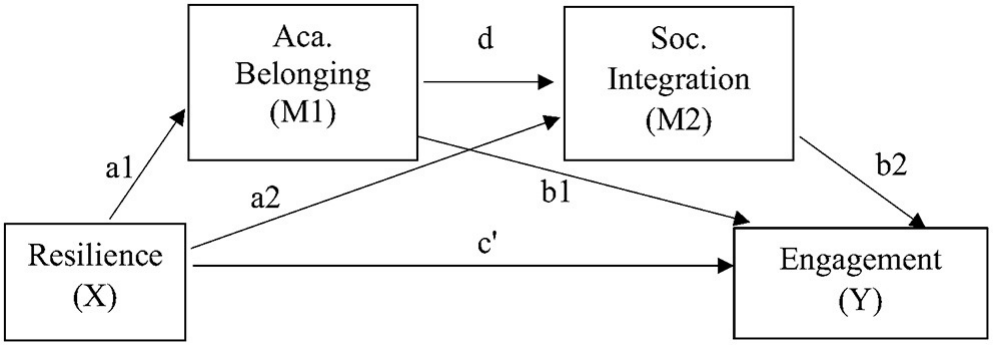
Explanatory serial mediation model with hypothesized direct pathway, independent mediation pathways, and serial mediation pathway between engagement, resilience, academic belonging, and social integration. Aca, academic; Soc, social.

The hypothesized model explores the presence of a direct predictive effect of resilience on engagement, in addition to exploring the presence of indirect predictive effects from academic belonging, social integration, and a serial mediating effect between the two (Figure 1). More so, student groups were included based on resilience levels, so as to assess predictive model fit between groups. The extended study hypotheses can be viewed in detail in Box 1. To our knowledge, no studies to date have sought to analyse academic belonging, social integration, and engagement among resilience-based HE student groups during the COVID-19 pandemic and ERT. As such, the current study will serve to expand scientific insight, in addition to informing student engagement and well-being strategies during periods of emergency remote teaching in higher education.

Box 1 **Study hypotheses.**

- A direct predictive effect of resilience on engagement, where resilience positively predicts engagement.
- A mediation effect of academic belonging, where a higher sense of academic belonging has a stronger positive effect on engagement.
- A mediation effect of social integration, where a higher level of social integration has a stronger positive effect on engagement.
- A serial mediation effect where the indirect effect of academic belonging on social integration predicts a positive effect on engagement.

## Method

### Survey

Between May 17th and May 22nd, 2021, students were invited to complete the Student Well-being Monitor 2021 (SWM 2021). Invitations were sent via email, and the survey was completed online via Qualtrics survey tool in accordance with European guidelines on General Data Protection Regulation (GDPR). The study was approved on ethical standards as defined by the institutional review board at Inholland University of Applied Sciences.

The SWM 2021 entails an extensive survey to assess well-being and student success via multiple broad domains including concerns and worries centered around the COVID-19 pandemic, the experienced changes due to the ERT transition, experience of study-related burn-out symptoms, engagement and resilience, and aspects of study behavior and integration. All domains and items included in the SWM 2021 are available elsewhere (46).

In the Netherlands, the HE system offers two distinct forms of higher education. The first includes an academic research oriented higher education offered by research universities (in Dutch: wetenschappelijk onderwijs), whereas the second entails higher professional education offered by universities of applied sciences (in Dutch: hoger beroepsonderwijs). The current study focused exclusively on participants from universities of applied sciences.

### Participation

Participation was voluntary and participants were invited to partake in the study if they were currently enrolled and were aged 17 years or older, and were studying at Inholland University of Applied Science. Consent was required prior to proceeding. All participants who had successfully completed the survey were included in the dataset.

### Measurements

#### Engagement

Student engagement was measured with the ultra-short Utrecht Work Engagement Scale-Student Form (UWES-3SF), a self-reported three-item scale with acceptable reliability and validity for use among HE students (30–32). The questionnaire required respondents to indicate occurrence frequency along a 7-point scale ranging from ‘never’ (1) to ‘always’ (7). The UWES-3SF captures student engagement along three subdomains, namely vigor, dedication, and absorption. Items include the statements ‘I have an abundance of energy when I study’, ‘I am enthusiastic about my study’, and ‘I am completely absorbed by my study’. Total scores were averaged with higher scores indicating a higher level of engagement. Cronbach's alpha for the UWES-3SF was .822.

#### Resilience

Resilience was measured using the Brief Resilience Scale (BRS) which is a short, self-reported 6-item measure of which validity and reliability has been assessed in other cohort studies (56). An indication of agreement with the statements was required according to a 5-point Likert scale, ranging from ‘total disagreement’ (1) to ‘total agreement’ (5). BRS items include ‘I tend to bounce back quickly after hard times’, ‘I have a hard time making it through stressful events’, and ‘it does not take me long to recover from a stressful event’. Four items had to be reversed prior to summing and averaging scale scores. BRS summed mean scores can be grouped to classify resilience as low, normal, or high using mean scores and standard deviations (66). Within the sample, the Cronbach's alpha value of the BRS was .872.

#### Academic Belonging

With available literature emphasizing a need for clearly defined constructs (21, 34, 35, 67, 68), this study developed and evaluated a short scale of academic belonging. Based on available questionnaires on behavioral, emotional, extracurricular, academic, and social engagement within the HE study context (69–71), relevant items were selected by the authors for analyses. Subsequently, items were assessed to scrutinize content validity, ambiguity, and applicability within online educational settings. Finally, items were analyzed using oblique factor analysis, reliability analysis, and Pearson's moment-product correlation testing to confirm valid and reliable subscale use (72).

The analyses revealed that academic belonging could be measured using a 6-item scale (Table 1). The items included statements such as ‘I feel like I can be myself within this study’, ‘my teachers know me’, and ‘teachers make sure students feel safe to ask questions’, all of which required responses along a 5-point Likert scale. Response categories ranged from ‘strong disagreement’ (1) to ‘strong agreement’ (5). Summed mean scores were calculated with higher scores indicating a higher sense of academic belonging. Reliability analysis of the scale demonstrated a Cronbach's alpha of .837.

**Table 1:** Scale assessment using Oblimin principal components analysis with Kaizer Normalization and Pearson's moment product correlation coefficient analysis for items and scale constructs of academic belonging.

**Item**			**Component loadings**				
			**1**	**2**	**3**	**4**	**5**
I feel at home at this study			0.735	0.102	−0.132	0.210	0.248
I feel like I can be myself within this study			0.713	0.054	−0.154	0.182	0.220
My teachers know me			0.513	0.087	−0.060	0.021	−0.307
Teachers are committed to their students			0.673	−0.051	−0.016	−0.047	−0.314
Teachers make sure students feel safe to ask questions			0.764	−0.058	0.026	−0.036	−0.133
Teachers are receptive to suggestions and feedback for improvements			0.778	−0.012	0.111	−0.146	−0.066
I participate in thinking about, and discussing, ways to improve education			−0.050	0.542	−0.108	0.065	−0.316
I commit myself to the higher education institute			−0.022	0.733	−0.035	−0.083	−0.109
I participate in extra (online) activities provided by my study			0.029	0.760	0.039	0.097	0.088
I participate in (online) social activities that are hosted by my study or study association			0.028	0.779	−0.022	−0.062	0.131
I am committed to my fellow students			−0.025	0.152	−0.733	0.095	−0.069
I can share my emotions and stories with fellow students			0.065	−0.032	−0.777	−0.010	−0.073
Being in touch with my fellow students helps me to perform well			0.061	−0.008	−0.752	−0.097	−0.002
I approach fellow students to work together on (online) assessments			−0.096	0.027	−0.737	0.099	−0.060
I work hard to succeed in my studies and spend a sufficient amount of time			−0.050	−0.002	−0.033	0.837	0.050
Usually, I participate in all study activities			0.122	0.015	−0.070	0.690	−0.004
I am rarely behind with the coursework for my study			−0.011	−0.045	−0.012	0.781	−0.051
I do not regularly do other things during class (e.g., Whatsapp, Facebook)			−0.046	0.144	0.293	0.357	−0.273
On occasions I discuss personal matters with teachers			0.113	0.167	−0.124	−0.085	−0.655
I know the names of the teachers whose classes I follow			0.048	−0.106	−0.126	0.201	−0.552
I discuss gained insights with teachers			0.120	0.080	−0.096	0.106	−0.655
Keeping in contact with teachers has a positive effect on my results			0.439	0.006	−0.059	−0.037	−0.453
**Pearson's moment product cross–correlations**							
**Academic belonging (6 items)**	**1**.	**2**.	**3**.	**4**.	**5**.	**6**.	**Scale**
1. I feel at home at this study	(–)	0.676^***^	0.431^***^	0.405^***^	0.404^***^	0.344^***^	0.720^***^
2. I feel like I can be myself within this study	.	(–)	0.460^***^	0.395^***^	0.404^***^	0.322^***^	0.716^***^
3. My teachers know me	.	.	(–)	0.509^***^	0.425^***^	0.360^***^	0.726^***^
4. Teachers are committed to their students	.	.	.	(–)	0.604^***^	0.589^***^	0.792^***^
5. Teachers make sure students feel safe to ask questions	.	.	.	.	(–)	0.621^***^	0.774^***^
6. Teachers are receptive to suggestions and feedback for improvements	.	.	.	.	.	(–)	0.731^***^
**Social Integration (4 items)**			**1**.	**2**.	**3**.	**4**.	**Scale**
1. I am committed to my fellow students			(–)	0.586^***^	0.440^***^	0.477^***^	0.792^***^
2. I can share my emotions and stories with fellow students			.	(–)	0.494^***^	0.448^***^	0.819^***^
3. Being in touch with my fellow students helps me to perform well			.	.	(–)	0.422^***^	0.748^***^
4. I approach fellow students to work together on (online) assessments			.	.	.	(–)	0.760^***^

^*^significant at p < 0.05.

^**^significant at p < 0.01.

*** *significant at p < 0.001*.

#### Social Integration

To assess social integration, four out of thirteen original items from the Social Integration subscale were used (69). The four items were selected based on applicability within the ERT context and ‘Cronbach's alpha if item deleted’. Implications of this selection process are further described in the limitations. Subsequently, an oblique principal components analysis was run to assess factor loadings, in addition to analyzing reliability and construct validity (Table 1). Results indicate that the reduced number of items taken from the original social integration subscale could assess social integration in the student sample. Items required participants to assess their level of agreement with the statements along a 5-point Likert scale, ranging from ‘strong disagreement’ (1) to ‘strong agreement’ (5). The items included ‘I can share my emotions and stories with fellow students’, and ‘I approach fellow students to work together on (online) assignments’. Average scores were calculated, with higher scores indicating higher social integration. The 4-item scale demonstrated a Cronbach's alpha of .784.

### Statistical Analysis

IBM SPSS Statistics for Windows, Version 27.0 was used to carry out analyses. The extension Macro PROCESS (73) version 3.5 was used to test model fit of a serial mediation effect of academic belonging and social integration on the direct effect of resilience on engagement. Bootstrapping techniques used in Macro PROCESS are robust against violations of normality by using confidence intervals to assess effect significance (74, 75), so no data transformations are required. Bootstrap resampling value was set at 5,000. To assess *post-hoc* power probabilities for the student groups, G^*^Power software version 3.1.9.6 was used (76).

A serial mediation analysis was conducted to estimate effect sizes and model fit for four groups: (1) all HE students, (2) students who reported low levels of resilience, (3) students who reported normal levels of resilience, and (4) students who reported high levels of resilience during the COVID-19 pandemic. During each analysis, the nature of the relationship between resilience and engagement (X and Y) was assessed directly, in addition to testing the indirect effect resulting from the two mediators academic belonging and social integration (M1 and M2), as well as their indirect serial mediating effect (Figure 1). The analytical workflow was derived from previous methods where multiple mediation analysis is based on two conditions. First, an examination is made to conclude whether the set of mediators transmits the effect of X to Y, and second, the specific indirect effect associated with each presumed mediator is tested. Within this framework, total indirect effects need not be significant for identification of relevant specific indirect effects (74).

Total, direct, indirect, and partial effects included in the model were described as statistically significant when the corresponding 95% confidence interval of the unstandardized effect size coefficient *b* did not contain zero. If the direct path between X and Y (c') was significant, and all three indirect pathways (a1 x b1; a2 x b2; and a1 x d x b2) yielded significant results, a partial serial mediation model is present. If the c' path effect between X and Y is non-significant and the three indirect pathways are significant, a full serial mediation model is present. If any of the indirect pathways fail to reach significance, the remaining partial pathways were examined. Each of the pathways was tested by regressing the corresponding variables. If the *b* coefficient of the estimated direct, serial indirect, or independent indirect effects occurred within a 95% confidence interval range excluding zero, the null hypothesis of no significant predictive effect was rejected.

A covariate inspection was conducted to identify variables that should be controlled for during the model fit analyses. To identify these, relevant sociodemographic and study trajectory variables were included based on indications of associations to engagement in available literature (25, 29, 32). As such, age, gender, study year, living arrangements, and study domain were inspected to determine if they displayed significant correlations to the dependent variable engagement. Subsequently, significantly correlated variables were examined to determine correlations with the independent variables. If a significant correlation to the dependent variable was present without additional significant correlations to the independent variables, inclusion criteria as covariate were met (72).

## Results

### Sample

A total of 1,848 participants completed the SWM 2021 survey. Data homogeneity inspection revealed that enrolment status created a significant impact on the distribution of the dependent variable; *F* (2.1844) = 27.590, *p* < 0.001. *Post-hoc* contrasts indicated that fulltime enrolment was significantly different from other forms of enrolment. Furthermore, students who identified as gender ‘x’ included 19 individuals, which failed to meet sample size criteria (72). A significant effect of study year was also found, where students studying 5 years or longer demonstrated different academic performance outcomes; *F* (4.1842) = 81.148, *p* < 0.001. Language (Dutch or English) displayed no significant effect on the outcome variable. As a result, all fulltime HE enrollers, studying for no more than 4 years, who identified as male or female were included in the final sample. The final sample contained 1,332 students of mean age 21.62 years (SD = 3.162). Of the respondents 481 were male (36.1%), and the remaining 851 were female (63.9%). The sample is described in further detail in Table 2.

**Table 2:** Sociodemographic characteristics of participants (*N* = 1,332).

	***N***	***%***
**Age in years**		
<18	33	2.5
18–20	481	36.1
21–23	563	42.3
24–27	200	15.0
>28	55	4.1
**Gender**		
Male	481	36.1
Female	851	63.9
**Study year**		
First	397	29.8
Second	397	29.8
Third	289	21.7
Fourth	249	18.7
**Living arrangements**		
Living at home with parent(s) or guardian(s)	981	73.6
Living independently with(out) roommates	351	26.4
**Language status**		
Dutch	1,174	88.1
English	158	11.9
**Study domain**		
Agri, food and life science	100	7.5
Business, finance, and law	310	23.3
Creative business	301	22.6
Health, sport, and well-being	307	23.0
Education and Innovation	79	5.9
Engineering, design & IT	235	17.6

No missing data was detected. More so, no outliers were identified as all variables were measured using Likert-scale responses. The final dataset was screened for violations that would prevent accurate use of Macro PROCESS. A Shapiro-Wilk normality test revealed non-normal data (Shapiro-Wilk statistic = 0.988, *p* < 0.001). However, the bootstrapping techniques used in Macro PROCESS are robust against violations of normality (74, 75), so no data transformations were needed.

Average scores of the final sample are displayed in Table 3. Calculation of low, normal, and high resilience student groups utilized the sample mean and standard deviation for all HE students (M = 2.894, SD = 0.793). As such, scores up to 2.101 were used to indicate ‘low’ resilience, scores ranging between 2.101 and 3.687 indicated ‘normal’ resilience, and scores above 3.687 were labeled as demonstrating ‘high’ resilience. 15.77% (*N* = 210) could be classified as having low resilience, 68.77% (*N* = 916) were classified as having normal resilience, and 15.47% (*N* = 206) reported a high level of resilience. *Post-hoc* examination of statistical power demonstrated sufficient detection power for all HE students groups (73). The power coefficient to detect small effect sizes was 1.000 for all HE students and the group of students with normal resilience. Among low resilience students the power coefficient was 0.998, and for high resilience students it came to 0.997.

**Table 3:** Descriptive statistics and Pearson's correlations of engagement, resilience, academic belonging, and social integration measures for all HE students and resilience groups.

		**M**	**SD**	**1**	**2**	**3**	**4**
**All students (*****N*** **=** **1,332)**						
1.	Engagement	3.750	1.248	(-)	0.134^***^	0.405^***^	0.286^***^
2.	Resilience	2.894	0.793	.	(-)	0.217^***^	0.143^***^
3.	Academic belonging	3.426	0.739	.	.	(-)	0.414^***^
4.	Social integration	3.372	0.813	.	.	.	(-)
**Low resilience (*****N*** **=** **210)**						
1.	Engagement	3.460	1.257	(-)	0.105^*^	0.398^***^	0.364^***^
2.	Resilience	1.732	0.302	.	(-)	0.216^**^	0.188^**^
3.	Academic belonging	3.205	0.823	.	.	(-)	0.452^***^
4.	Social integration	3.148	0.884	.	.	.	(-)
**Normal resilience (*****N*** **=** **916)**						
1.	Engagement	3.740	1.221	(-)	0.010	0.373^***^	0.245^***^
2.	Resilience	2.881	0.461	.	(-)	0.120^***^	0.081^**^
3.	Academic belonging	3.425	0.696	.	.	(-)	0.384^***^
4.	Social integration	3.397	0.770	.	.	.	(-)
**High resilience (*****N*** **=** **206)**						
1.	Engagement	4.070	1.286	(-)	0.125^*^	0.456^***^	0.299^***^
2.	Resilience	4.134	0.329	.	(-)	0.176^**^	0.006
3.	Academic belonging	3.660	0.764	.	.	(-)	0.415^***^
4.	Social integration	3.494	0.883	.	.	.	(-)

* *significant at p < 0.05*,

** *significant at p < 0.01*,

*** *significant at p < 0.001*.

### Covariates

Covariate analyses demonstrated that study year and living arrangement were the only variables with a significant correlation to engagement (age: *r* = −0.014, *p* = 0.607; gender: *r* = 0.034, *p* = *0*.213; study domain: *r* = 0.029, *p* = 0.289; study year: *r* = −0.165, *p* < 0.001; living arrangement: *r* = −0.070, *p* = 0.011). More so, no significant correlations were found between either study year, or living arrangements, and the independent variables. Therefore, study year and living arrangement were controlled as covariates in the subsequent model analyses.

### All Students

**Figure 2:**
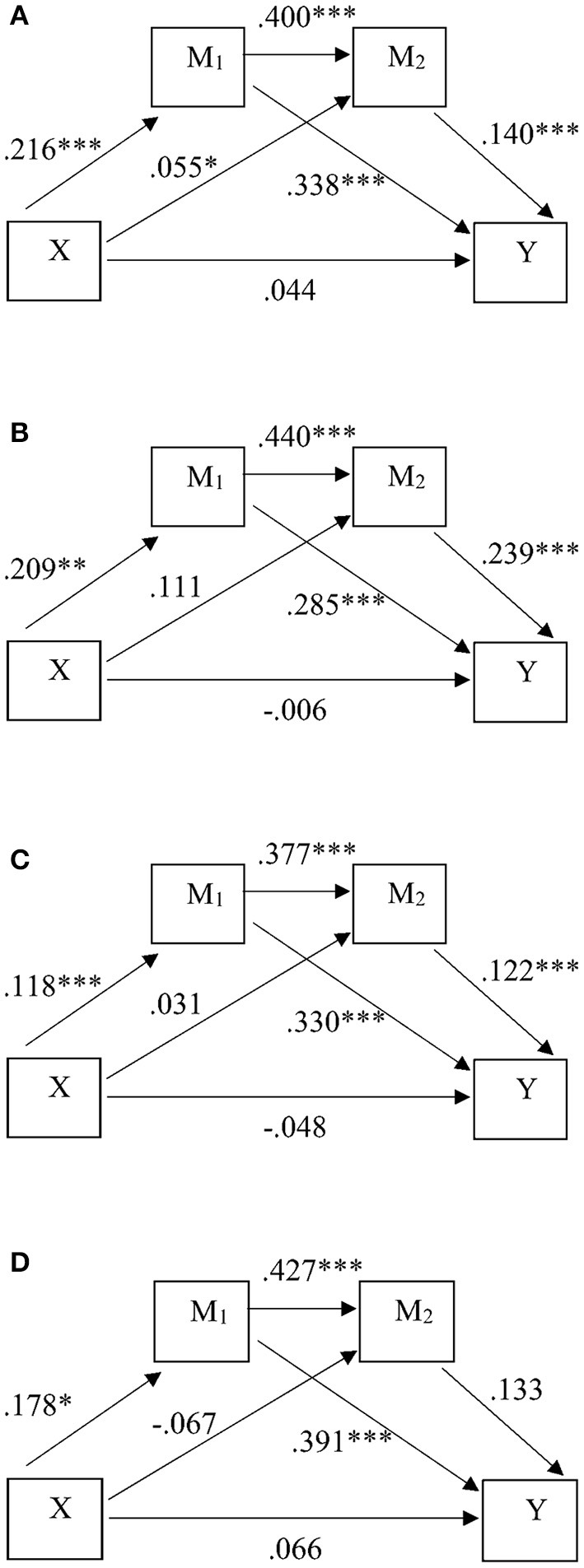
Predictive model standardized effects for the HE student groups: **(A)** all students (*N* = 1,332), **(B)** students with low resilience (*N* = 210), **(C)** students with normal resilience (*N* = 916), and **(D)** students with high resilience (*N* = 206). * significant at *p* < *0.05*, ** significant at *p* < *0.01*, *** significant at *p* < *0.001, X, resilience; Y, engagement; M1, academic belonging; M2, social integration*.

For all HE students, the total predictive effect of the hypothesized model was 0.137 (Tables 3, 4), with indirect effects accounting for 67.79% of the total effects. Furthermore, the *R*^2^ indicates an explained variance of 21%, suggesting moderate and adequate model fit (77). For both academic belonging and social integration, higher levels of resilience predicted higher levels of the mediators, which in turn predicted higher engagement (Figure 2A). Results demonstrate a full serial mediation model for all HE students' engagement, where resilience, academic belonging, and social integration are significant positive predictors of engagement, which aligns with the research expectations.

**Table 4:** Standardized (B) and unstandardised (b) regression coefficients, and significance tests for the explanatory model pathways between the HE student group and resilience groups.

**Pathway**	**B**	***b***	***t***	***p***	***R*** **^2^**	**95% CI**
**All students (*****N*** **=** **1,332)**					0.21	
a1	0.216	0.201	8.051	<0.001^***^		0.152 to 0.250
a2	0.055	0.056	2.137	0.033^*^		0.005 to 0.108
b1	0.338	0.570	12.409	<0.001^***^		0.480 to 0.661
b2	0.140	0.214	5.198	<0.001^***^		0.133 to 0.295
d	0.400	0.441	15.663	<0.001^***^		0.386 to 0.496
c'	0.044	0.069	1.755	0.080		−0.008 to 0.147
Cov. 1	−0.165	−0.190	−6.664	<0.001^***^		−0.246 to −0.134
Cov. 2	−0.020	−0.056	−0.790	0.430		−0.193 to 0.082
**X on Y**	**Effect**		**se**	***t***	***p***	**95% CI**
Total	0.137		0.042	5.092	<0.001^***^	0.132 to 0.298
Ind. total Ind1 (a1 x b1) Ind2 (a2 x b2) Ind3 (a1 x d x b2)	0.093 0.073 0.008 0.012		0.014 0.012 0.004 0.003			0.066 to 0.120 0.051 to 0.097 0.0003 to 0.0165 0.007 to 0.019
**Low resilience (*****N*** **=** **210)**					0.21	
a1	0.209	0.568	3.044	0.003^**^		0.200 to 0.936
a2	0.111	0.323	1.743	0.083		−0.042 to 0.688
b1	0.285	0.435	4.003	<0.001^***^		0.221 to 0.649
b2	0.239	0.339	3.369	<0.001^***^		0.141 to 0.538
d	0.440	0.472	6.983	<0.001^***^		0.339 to 0.606
c'	−0.006	−0.025	−0.091	0.928		−0.555 to 0.506
Cov. 1	−0.057	−0.066	−0.897	0.371		−0.212 to 0.080
Cov. 2	−0.073	−0.203	−1.135	0.258		−0.555 to 0.149
**X on Y**	**Effect**		**se**	***t***	***p***	**95% CI**
Total	0.114		0.289	1.462	0.145	−0.147 to 0.994
Ind. total Ind1 (a1 x b1) Ind2 (a2 x b2) Ind3 (a1 x d x b2)	0.108 0.059 0.026 0.022		0.039 0.029 0.018 0.012			0.036 to 0.187 0.011 to 0.125 −0.005 to 0.065 0.004 to 0.049
**Normal resilience (*****N*** **=** **916)**					0.19	
a1	0.118	0.178	3.582	<0.001^***^		0.081 to 0.276
a2	0.031	0.052	1.017	0.310		−0.049 to 0.153
b1	0.330	0.579	10.194	<0.001^***^		0.467 to 0.690
b2	0.122	0.193	3.770	<0.001^***^		0.093 to 0.294
d	0.377	0.417	12.290	<0.001^***^		0.351 to 0.484
c'	−0.048	−0.128	−1.607	0.108		−0.285 to 0.028
Cov. 1	−0.193	−0.219	−6.329	<0.001^***^		−0.287 to −0.151
Cov. 2	−0.034	−0.094	−1.099	0.272		−0.262 to.074
**X on Y**	**Effect**		**se**	***t***	***p***	**95% CI**
Total	0.097		0.086	0.003	0.997	−0.169 to 0.169
Ind. total Ind1 (a1 x b1) Ind2 (a2 x b2) Ind3 (a1 x d x b2)	0.048 0.039 0.004 0.005		0.014 0.011 0.004 0.002			0.023 to 0.075 0.018 to 0.062 −0.004 to 0.013 0.002 to 0.011
**High resilience (*****N*** **=** **206)**					0.25	
a1	0.178	0.412	2.541	0.012^*^		0.092 to 0.731
a2	−0.067	−0.178	−1.022	0.308		−0.526 to 0.167
b1	0.391	0.658	5.714	<0.001^***^		0.431 to 0.886
b2	0.133	0.193	1.968	0.050		−0.003 to 0.387
d	0.427	0.493	6.570	<0.001^***^		0.346 to 0.642
c'	0.066	0.258	1.051	0.295		−0.226 to 0.741
Cov. 1	−0.157	−0.180	−2.561	0.011^*^		−0.318 to−0.041
Cov. 2	0.053	0.152	0.855	0.393		−0.199 to 0.503
**X on Y**	**Effect**		**se**	***t***	***p***	**95% CI**
Total	0.137		0.271	1.968	0.050	−0.001 to 1.067
Ind. total Ind1 (a1 x b1) Ind2 (a2 x b2) Ind3 (a1 x d x b2)	0.071 0.069 −0.009 0.010		0.044 0.038 0.011 0.007			−0.013 to 0.164 0.003 to 0.150 −0.032 to 0.010 −0.001 to 0.025

*a1, independent variable (IV) to mediator 1; a2, IV to mediator 2; b1, mediator 1 to dependent variable (DV); b2, mediator 2 to DV; d, mediator 1 to mediator 2; c', IV to dependent variable; Cov.1, study year control variable; Cov.2, living arrangement control variable*;

* *significant at p < 0.05*;

** *significant at p < 0.01*;

*** *significant at p < 0.001*.

Of the indirect effects, the strongest predictive effect involves the pathway via academic belonging, at 78.73% of total indirect effect, with effect size 0.073. The indirect effect via social integration was smaller at 8.21% of the total indirect effects, whereas the serial mediating indirect effect was 13.07% of the total indirect effects. In addition, results display positive partial effects between academic belonging and social integration, and from academic belonging on engagement, with effects classified as large and very large (78).

### Students With Low Resilience

Students with low levels of resilience also reported the lowest levels of academic belonging, social integration, and engagement (Table 3). More so, total predictive effects of the model did not reach significance, although the *R*^2^ value indicates moderate and adequate model fit (77) at 0.20 (Table 4). Of the total effects, 94.72% originated from significant indirect effects via independent and serial mediation of academic belonging and social integration. Of the indirect effect total, 55.20% stemmed from the significant indirect pathway through academic belonging, and 20.35% came from the significant serial mediation effect of both mediators.

No significant indirect effect via social integration was found, even though the partial predictive effect from social integration on engagement was largest compared to other student groups (compared to all students: +170%, compared to normal resilience: +220%, compared to high resilience: +180%) (Figure 2B). Furthermore, compared to other groups, low resilience students display the smallest partial effect of academic belonging on engagement, at *B* = 0.285 (compared to all students: −15.68%, compared to normal resilience: −13.64%, compared to high resilience: −27.11%).

In contrast to research expectations, a larger serial mediation effect was present at an effect size of 0.022 (compared to all students: +183%, compared to normal resilience: +440%, compared to high resilience: +220%). Findings demonstrate the presence of serial mediation via both academic belonging and social integration and additionally indicate that students with low resilience show the strongest serial predictive effects compared to the other student groups. In addition, the partial predictive effect from social integration on engagement was largest compared to other student groups which contrasted expected outcomes. In line with expectations, results did indicate a smaller partial effect of academic belonging on engagement for students with low resilience.

### Students With Normal Resilience

Students with normal resilience levels scored in the middle range regarding engagement, academic belonging, and social integration in comparison to low and high resilience groups (Table 3). Concerning predictive model outcomes, although the model fit is moderate and adequate (77) and explains 19% of the variance, the total effects are insignificant (Table 4). In addition, the relationship between X and Y was insignificant at *r* = 0.010 (Table 3), indicating the absence of a direct association between resilience and engagement. Consequently, no mediation model can be seen among this group, with results instead indicating predictive indirect and partial effects (Figure 2C).

The total indirect effects were significant, although, at 0.048, this effect size was smallest compared to the other student groups. Of the indirect effects, 81.25% originated from the indirect effect via academic belonging, and 10.42% originated from the serial effects via both mediators. The serial effect size was also smallest for this group compared to the other groups (compared to all students: −58.33%, compared to low resilience: −77.27%).

Of the significant partial effects, three effects were smallest compared to the other student groups. The partial effect of resilience on academic belonging displayed a small effect (78), which was lowest compared to other groups (compared to all students: −45.37%, compared to low resilience: −43.54%, compared to high resilience: −33.71%). The predictive serial effect between the two mediators was also smallest compared to the other groups (compared to all students: −5.75%, compared to low resilience: −14.32%, compared to high resilience: −11.71%). The partial effect from social integration to engagement was again smallest compared to other groups, with the decrease ranging between 20.00% and 53.14%. These results misalign with research expectations, as a direct predictive effect of resilience on engagement was absent amongst this group. Instead, results indicate an indirect predictive effect via academic belonging and a serial indirect effect via both academic belonging and social integration on engagement. Additionally, results contrast expectations concerning effect sizes compared to other student groups with the group of students with normal resilience displaying predictive effects that are smaller compared to other groups.

### Students With High Resilience

The students with high resilience reported the highest levels of academic belonging, social integration, and engagement compared to other student groups (Table 3). Furthermore, the analysis revealed that the model explained 26% of the variance, indicating adequate and substantial fit (77). The total effects of the model were marginally insignificant at *p* = 0.050 (Table 4). Further inspection revealed that the direct pathway between resilience and engagement, the indirect pathway through social integration, and the serial mediation pathway were all insignificant (Figure 2D). The only significant pathway involved an indirect mediation effect via academic integration, where higher resilience predicts higher academic belonging and this subsequently predicts higher engagement, at 97.18% of the total indirect effects. At an effect size of 0.069, this indirect mediation effect is larger compared to other student groups (compared to low resilience: +14.49%, compared to normal resilience: +43.48%). Although a very large partial effect (78) was revealed between the two mediators at 0.427, no subsequent predictive partial effect on engagement was indicated. These findings partly contrast research expectations as serial mediation was absent, but also partially aligned with expectations as the partial effect of academic belonging on engagement was largest compared to low and normal resilience groups.

## Discussion

This study researched engagement predictors among HE students studying via ERT in the Netherlands during the COVID-19 pandemic. The relationship between resilience and engagement was examined, in addition to assessing mediating and indirect effects via academic belonging and social integration. For all HE students, serial mediation was demonstrated where the direct effect of resilience on engagement was fully mediated by academic belonging and social integration. Resilience positively predicts academic belonging, which in turn positively predicts engagement and social integration. Resilience additionally positively predicts social integration with fellow students, which in turn positively predicts engagement. Moreover, independent partial predictive effects from academic belonging and social integration on engagement were found. As such, findings aligned with research expectations for the entire HE student sample.

The confirmation of a significant relationship between resilience and engagement during ERT, lines up with findings regarding face-to-face teaching (24, 49). Among all students, mediation by both academic belonging and social integration are in keeping with studies on the positive mediating role of feeling at home at the HEI and positive peer relationships on engagement (29, 35). Distinct from studies on engagement during face-to-face teaching, the current study reveals that engagement during ERT was predicted with greatest effect through academic belonging (42–44).

Results also displayed serial mediation among low resilience students, with the largest effect size of all student groups in contrast to expectations. The indirect predictive effect from academic belonging on engagement contained the largest predictive effect, although this effect was smaller relative to the other groups. Furthermore, independent mediation via social integration was absent, even though the partial predictive effect was largest compared to other student groups.

These findings suggest several points. First, students with low resilience experience lower levels of belonging at their HEIs during ERT, and it stimulates engagement to a lesser extent than among other student groups. Students with lower resilience may thus be more prone to feeling unsafe within the academic context, or may experience lower connectedness to their educational programs, both under face-to-face teaching circumstances and during ERT (79–81), potentially indicating a characteristic of low resilience students. Low resilience students may also experience more difficulty expressing their needs and questions within the academic setting, limiting the positive effect on engagement compared to other groups.

Second, low resilience students reported the lowest mean levels of social integration, whilst simultaneously demonstrating the largest partial effects related to social integration. This indicates the presence of lower positive relationships with fellow students during ERT, while depending on social integration to facilitate engagement to a higher degree than the other student groups. Given the suboptimal quality of social integration during ERT (47), engagement for this group could be improved by enhancing social integration.

Third, as the largest relative serial mediation effect was found among low resilience students, this group utilizes this indirect pathway to a larger extent. As such, targeting academic belonging among low resilience students could include a two-hit approach during ERT: it could facilitate engagement directly, and it could increase social integration, subsequently enhancing engagement.

Finally, promoting resilience among low resilience students could prove a promising strategy. Based on results from the other student groups, increasing resilience could enhance students' sense of academic belonging during ERT, which is particularly relevant during the changing educational contexts related to the COVID-19 pandemic. More so, with resilience so prominently linked to maintained wellbeing during the pandemic (57), targeting HE students with suboptimal resilience could provide positive benefits to wellbeing that extend beyond the realm of the educational setting (55).

When students reported normal levels of resilience the direct association between resilience and engagement ceased to be found, which misaligned with the study's expectations. Instead, students with normal resilience exhibited significant indirect effects, including a serial indirect effect via academic belonging and social integration that was smallest compared to the other groups. The sense of academic belonging during ERT displayed the strongest indirect predictive effect on engagement, although it was ultimately smaller compared to other groups, due to the relative smaller effect from resilience. This suggests that resilience had a lesser effect on engagement maintenance among normally resilient students.

Furthermore, the explained variance of the model was lowest, suggesting application of alternative engagement strategies among these students. With previous research indicating that intrinsic values such as motivation, desire to succeed, determination, and future orientation are present amongst resilient individuals (82, 83) our findings may indicate that this student group is using intrinsic factors excluded from the current study design. They may also be utilizing alternate engagement resources, as higher resourcefulness is also instrumental to engagement (49).

For highly resilient students, serial mediation was absent and social integration failed to predict engagement during ERT. Instead, indirect mediation through academic belonging alone demonstrated the strongest predictive effect relative to other groups. Highly resilient students' sense of academic belonging displayed the highest average scores, the largest indirect effect, and the largest partial predictive effect on engagement, suggesting a superior sense of belonging within a limited ERT higher educational context. This outcome aligns with studies indicating higher levels of connectedness among highly resilient students under face-to-face educational conditions (55) and aligns with increased adaptability among students with higher engagement during ERT (6).

Additionally, none of the predictive pathways associated with social integration were significantly present among highly resilient students, even though these students reported the highest levels of social integration during ERT. These findings differ from previous indications of advanced utilization of peer student support among highly resilient students (82) and contrast findings for low resilience students. The current model may have captured a context driven adaptation, where, given the limitations of social integration under ERT, highly resilient students have shifted engagement tactics away from social integration. After all, with social integration potentially hindered during ERT if fellow students do not participate (18), our findings could reflect stronger application of self-controlled strategies among this student group.

Our explanation lines up with research demonstrating increased positive adaptability among more engaged students (6) and expands on studies indicating that HE students report an increased need for self-discipline, motivation, self-teaching skills, and organization to successfully maintain learning and engagement during ERT (18). Thus, although highly resilient students are reporting social relationships and collaborations with fellow students to a higher level than other student groups, they boost engagement during ERT primarily through their heightened sense of academic belonging.

### Limitations

This study has some limitations. First, operationalization of engagement can be highly variable between studies, limiting comparability (67, 68). Though the current study included an engagement scale that has been used previously in global cohort studies (23, 28–33), comparability to alternate engagement scales depends on subcomponent overlap and construct definition. The UWES-SF does not focus on behavioral components of engagement, which limits comparability of findings to cognitive-affective engagement studies. Second, although the current study included sociodemographic and study related covariates to control their independent influence on the outcome variable, unincluded variables such as social economic status, family social support, or family educational background may also be relevant. Future studies should include additional sociodemographic variables to allow control of covariance. Third, initial selection of a subset of items from the social integration scale lacked a primary factor analysis to test validity. Though the entire scale has been validated in a sample of HE students (51) the subset was not, which could have influenced validity optimization of the current subscale. Future research should establish validity of reduced scales to validate utilization of short forms. Finally, based on available literature indicating directional associations, the current serial mediation model assumes the presence of predictive effects between included constructs. However, studies also indicate alternative associations between these constructs (38, 84) including feedback effects, which are not captured in the current model. More so, assessment of directionality is limited in cross-sectional datasets, as opposed to longitudinal monitoring or experimental designs. Further research should focus on examining additional aspects relevant to engagement and resilience, and should further clarify directional processes by using longitudinal approaches or interventions.

### Practical Implications and Future Directions

For all HE students who are studying via ERT in the Netherlands, interventions geared toward stimulation of resilience, academic belonging, and social integration all stand to provide significant engagement benefits. The current study also underlines the need for individualized, profile-oriented approaches, as engagement interventions might affect HE students differently depending on their resilience profiles. HEIs should consider student resilience characteristics to assess which intervention targets are promising among their student populations.

Students with low resilience could benefit more from interventions aimed at improving the level of student interactions with fellow students, increasing academic belonging, as well as increasing resilience. Highly resilient students on the other hand, stand to profit most from interventions aimed solely at raising academic belonging, whereas students with normal resilience would be supported by interventions targeting academic belonging and social integration, but not resilience.

The current study provides several important directions for future research. Regarding different resilience profiles, future studies should expand current knowledge by continuing examination of predictive models while ERT endures and once face-to-face teaching at HEIs re-opens. In doing so, predictive stability among different resilience groups can be analyzed, and adaptability to shifts between ERT and face-to-face teaching in HEIs can be assessed.

In addition, incorporation of other relevant aspects of engagement, including measures of determination, self-motivation, and organization skills will help determine to what extent such tactics are also pivotal to engagement maintenance. Examining these constructs will diversify applicable intervention targets that HEIs could utilize to support HE students' engagement levels during ERT. Finally, future research on student engagement should further focus on risk factors and protective influences on engagement among students with low resilience. As these students display the lowest levels of academic belonging, social integration, and engagement, this group could be more at risk of slipping through the cracks during ERT. As such, continued assessment of student groups with higher risk profiles is warranted to ensure prevention, early signaling, and timely support.

Inspecting predictive models for HE students based on distinct resilience typologies offer new insights on intra-group differences and has been recognized previously as an important yet underrepresented area of research (29, 85). Overall, our study reveals resilience dependent changes in student engagement predictors during ERT in the Netherlands. In addition, the current study demonstrates model outcomes that contrast studies conducted in face-to-face higher educational settings, potentially reflecting impacts of ERT. As ERT is linked to limited interactions with peer students, lowered participation in class discussion, and a lack of instant feedback (79), HE students may well be reconfiguring how to best maintain engagement during this time. Current findings argue for continued research focussed on student resilience and engagement during ERT, especially given the potential recurrence of lockdown restrictions for HEIs in the Netherlands following identification of new COVID-19 variants (86).

## Data Availability Statement

The datasets presented in this article are not readily available because study participants did not consent to public availability of their data. Requests to access the datasets should be directed to rutger.kappe@inholland.

## Ethics Statement

The studies involving human participants were reviewed and approved by the Institutional Review Board Inholland University of Applied Sciences. Online consent was required prior to study participation of students. Written informed consent from the participants' legal guardian/next of kin was not required to participate in this study in accordance with the national legislation and the institutional requirements.

## Author Contributions

MV contributed to the survey design, conducted the data analysis, and led the literature research and manuscript production. RK aided in survey design and contributed to the literature research. CK contributed to survey design, scale development, and literature research. All authors contributed to the article and approved the submitted version.

## Funding

This work was supported by the Department of Education and Innovation, Research Group Study Success, Inholland University of Applied Sciences, Haarlem, Netherlands.

## Conflict of Interest

The authors declare that the research was conducted in the absence of any commercial or financial relationships that could be construed as a potential conflict of interest.

## Publisher's Note

All claims expressed in this article are solely those of the authors and do not necessarily represent those of their affiliated organizations, or those of the publisher, the editors and the reviewers. Any product that may be evaluated in this article, or claim that may be made by its manufacturer, is not guaranteed or endorsed by the publisher.
